# Determining the Antibacterial Effect of *Mentha Longifolia* Essential Oil on Cariogenic Bacteria and Its Compounds: an *in vitro* Study

**DOI:** 10.30476/dentjods.2022.92992.1688

**Published:** 2023-03

**Authors:** Fardokht Shazdehahmadi, Abazar Pournajaf, Sohrab Kazemi, Maryam Ghasempour

**Affiliations:** 1 Student Research Committee, Babol University of Medical Sciences, Babol, Iran; 2 Infectious Diseases and Tropical Medicine Research Center, Health Research Institute, Babol University of Medical Sciences, Babol, Iran; 3 Cellular & Molecular Biology Research Center, Health Research Institute, Babol University of Medical Sciences, Babol, Iran; 4 Oral Health Research Center, Health Research Institute, Babol University of Medical Sciences, Babol, Iran

**Keywords:** Dental caries, *Streptococcus mutans*, *Streptococcus sobrinus*, *Lactobacillus*

## Abstract

**Statement of the Problem:**

Continuous use of chemical agents to reduce the number of cariogenic bacteria leads to adverse effects; therefore, in recent years, many studies have focused on plant-based substances.

**Purpose:**

This study explores the antibacterial effects of *Mentha Longifolia* (*M. longifolia*) essential oil on *Streptococcus mutans* (*S. mutans*), *Streptococcus sobrinus* (*S. sobrinus*), and *Lactobacillus* as cariogenic microorganisms and determines the compounds in it.

**Materials and Method:**

In this experimental study, *S. mutans*, *S. sobrinus*, and Lactobacillus isolates were collected from the saliva samples of five children with severe early childhood caries (S-ECC). The minimum inhibitory concentration (MIC) and minimum bactericidal concentration (MBC) of *M. longifolia* essential oil were determined by Broth microdilution method. Chlorhexidine 0.2% and phosphate-buffered normal saline (pH= 7.0) were used as positive and negative controls, respectively. The chemical composition of *M. longifolia* essential oil was evaluated by gas chromatography-mass spectrometry. The data were analyzed with a paired t-test and the *p* below 0.05 was considered significant.

**Results:**

The MIC and MBC ratios for *S. mutans* were 3.12% and 6.25%, for *S. sobrinus* were 6.25% and 12.5%, ​​and *Lactobacillus* were 3.12% and 6.25%, respectively. Chemical analysis of *M. longifolia* essential oil showed that 34 various compounds. Piperitone oxide (27.59%), Transcariophylline (14.55%), and 2-cyclohexane-1-one (12.24%) were the major constituents.

**Conclusion:**

*M. longifolia* essential oil has both growth inhibitory and bactericidal effects on all the three species of bacteria. This antibacterial effect was similar against *S. mutans* and Lactobacillus, which was greater than *S. sobrinus*, thus, it can be used as a supplementary for caries prevention compounds.

## Introduction

Dental caries, an infectious microbial disease, is one of the most prevalent chronic diseases worldwide [ 1 ]. The disease requires a host (teeth in the oral environment), a nutrient medium, and bacteria. *Streptococcus mutans* (*S. mutans*), *Streptococcus sobrinus* (*S. sobrinus*), and *Lactobacilli* are human odontopathogens. *S. mutans* is the main and the most important cariogenic microorganism. *S. sobrinus* and *Lactobacillus* are also involved in later stages of the caries process [ 2 ]. Globally, caries prevalence is very high [ 1 ] and a more focused attention to preventive measures is necessary.

Reduction in the number of oral microorganisms prevents dental caries [ 3 ]. One of the most popular antibacterial chemical agents used in the mouth is chlorhexidine. The antimicrobial effect of chlorhexidine is due to its cationic property, which gives it persistent bactericidal and bacteriostatic effect on tooth surfaces. However, loss of taste sensation, teeth discoloration, burning sensation of the oral mucosa, and dry mouth has been reported as side effects of chlorhexidine [ 4 - 5 ]. Other popular antimicrobial agents, antibiotics, and antimicrobial mouthwashes have numerous side effects despite their usefulness [ 5 - 7 ], so the search for new antimicrobial agents with minimal side effects is warranted.

*Mentha Longifolia* (*M. Longifolia*) belongs to the *Lamiaceae* family. It grows mainly in the wild and in humid places such as riverbanks. It is found throughout the temperate regions of Central and Southern Europe, Southwest Asia, Australia, North Africa, Ethiopia, the Canary Islands, and on the slopes of Alborz Mountains, Northern, and Northeast Iran. It spreads to the East and some other places of Iran [ 8 ]. Due to its many biological properties, it has been studied extensively. Its medicinal properties include anti-inflammatory, anti-mutagenic, antioxidant, anti-rheumatic, antispasmodic, anti-viral, anti-candida, anti-platelet adhesion, muscle relaxant, and Cyclooxygenase inhibitor properties [ 9 - 12 ]. Some studies have evaluated the antimicrobial activity of the essential oil of this plant [ 12 - 13 ]. Hydrophobicity is one of the most important features of essential oils and accounts for their antimicrobial effect. This property enables the essential oils to penetrate the membrane lipids and mitochondria of bacteria, making the membrane more permeable and causing the release of ions and other intracellular contents of bacteria [ 12 ].

Studies have shown antibacterial properties of *M. longifolia* against many gram-positive and gram-negative bacteria [ 12 - 13 ]. In addition, several researches were done to evaluate the components of *M. longifolia* essential oil that reported different major compositions of the plant [ 14 - 15 ] and the composition depended on climatic or geographic conditions [ 16 ].

The aim of this study was to investigate the antibacterial effect of *M. longifolia* essential oil on oral cariogenic bacteria, such as *S. mutans*, *S. sobrinus*, and *Lactobacillus*. In addition to saliva sampling of children with severe early childhood caries (S-ECC) [ 17 ], standard strains were also used and the results were compared with chlorhexidine. In addition, components of essential oil were also investigated. 

## Materials and Method

This experimental study has been approved by Babol University of Medical Sciences with the code of ethics IR.MUBABOL.HRI.REC.1398.318.

### Plant collection

*M. longifolia (L.)* *Hudson* (*Lamiaceae*) aerial parts were harvested in the spring season in the valley of Opert (Mazandaran province, Iran). The collected plants were identified and confirmed by the Department of Botany, Sari Faculty of Agriculture (Iran).

For essential oil extraction, nine hundred and eighty grams of fresh *M. longifolia* aerial parts was chopped and the essential oil extracted through distillation and Clevenger method (Schottduran- Germany).

### Microbial tests

#### Preparation of bacterial strains

*S. mutans*, *S. sobrinus*, and *Lactobacillus* strains were collected from the non-duplicative saliva samples of five children [ 18 - 19 ] with S-ECC. Consent was obtained orally after justifying the dimensions of the research. Children in the study had no medical problems as well as not taking antibiotics, steroids, and topical fluoride therapy history in a month before sampling. In brief, 2 ml of unstimulated saliva was taken from each child. The children spotted the saliva, as instructed in 3 to 5minutes in sterile plastic containers with screws [ 20 ].

#### Detection of bacteria using specific tests

After transfer all saliva samples to the Department of Microbiology, Babol University of Medical Sciences (Iran), a 100μl of each sample was inoculated into the Blood agar, Mitis Salivarus Agar (MSA) and de Man, Rogosa and Sharpe (MRS) plates (Merck, Germany). The plates were incubated at a CO_2_ atmosphere in a 37° C for 24 hours. Suspected streptococcal colonies were identified by biochemical tests such as gram staining, mannitol and sorbitol fermentation, colin and arginine hydrolysis, catalase, and sensitivity to bacitracin whereas spore-free gram-positive bacilli, catalase-positive and negative indol test results were also performed to identify lactobacilli. To store the strains for a long time, all samples were cultured in brain heart infusion (BHI) broth containing 20% glycerol and kept at -70°C. *S. mutans* *(PTCC1683)*, *S. sobrinus* *(PTCC27607)* and *La-ctobacillus* *(PTCC1643)* were used as positive quality controls. 

#### Antimicrobial effect of *M. longifolia* essential oil

To compare the effect of *M. longifolia* essential oil on the tested bacteria, 0.2% chlorhexidine and phosphate-buffered normal saline (pH 7.0) were used as positive and negative quality controls, respectively.

#### Determination of minimum inhibitory concentration (MIC)

The minimum inhibitory concentration (MIC) of *M. longifolia* essential oil against the target bacteria was determined by broth microdilution method in 96 house plates. Serial dilutions of *M. longifolia* were prepared in completely sterile wells. First, 50 μl of the essential oil was poured into the first well using a sampler. Another 50 μl was taken from the first well into the second well in the same row. A 50 μl was taken from each preceding well and added to the succeeding well in the same row until well number 11. Serial dilutions of 50, 25, 12.5, 6.25, 3.12, 1.56, 0.78, 0.2 and 0.1µg/ml concentrations of *M. longifolia* were produced. Half McFarland's turbidity (1.5×10^8^CFU/ml) [ 21 ] was added to all wells. Then a medium containing 100 bacteria was added to each well. Well number 12 in each row contained 0.2% chlorhexidine, which served as a positive control. After inoculation of all the wells, the microplate was placed on a shaker for 30 seconds to achieve a uniform mixture. The mixture was incubated at 35°C for 24 hours in an anaerobic jar. The first well in which no growth was observed was designated as the MIC. The results were repeated three times.

#### Determination of minimum bactericidal concentration (MBC)

Ten microliter was removed from MIC dilution and a few higher dilutions and cultured on blood agar and MRS agar media. A concentration of essential oil in which no bacterial growth was observed on the medium was reported as MBC. The results were repeated three times.

#### Gas chromatography analysis of essential oil

Helium gas (with a purity of 99.999%) was injected into the column of a gas chromatography machine at a rate of 0.8 ml/min. The temperature of the column was raised from 40°C to 208°C at a rate of 5°C/min. 

Inhibition index (IR) for all components of *M. longifolia* essential oil was calculated by injecting syringes of nalkanes (C5-C25) according to the conditions of the samples. Components of the essential oil were identified by comparing the retention time and spectroscopic pattern of detected components with authentic standard retention time and spectroscopic patterns, respectively. 

#### Gas chromatography-mass spectrometry (GC – MS) of *M. longifolia* extracts

Phytochemical analysis of *M. longifolia* extracts was carried out using a G/C Agilent 5975 gas chromatography mass spectrometer (Agilent Company, United States). The gas chromatograph was set as follows: an HP-5-MS UI capillary column (30m×0.25mm; 0.25μm film thickness), a helium carrier gas with a flow rate of 1ml/ min, an oven temperature of 40°C and adjusted to 200° C at 5°C/min, an injection volume of 1μl, injector and detector temperatures of 280°C, and a split ratio of 10:1. 

Mass spectrometry has been adjusted with an ionization potential of 70 eV, a mass range of 40 to 550 amu, and a 2000 V energy electron multiplier. Identification of the phytochemical components of *M. longifolia* essential oil was determined by comparing the results of the GC-MS analysis with the reference retention time and spectral mass data of the NIST9 and wiley7 database [ 22 ].

### Data analysis

Data from the study were analyzed using the statistical software SPSS version 24.00. Paired Sample T-Test was performed for each group. *p* less than 0.05 was considered significant (*p*≤ 0.05).

## Results

### MIC and MBC test results for *M. longifolia* essential oil

According to Table 1, 0.2% chlorhexidine had the highest antibacterial effect on all the studied microorganisms compared to *M. longifolia* (*p* < 0.05).

**Table 1:** MIC and MBC levels of *Mentha longifolia* (*M. longifolia*) essential oil and chlorhexidine (CHX) on cariogenic microorganisms

Organism name	Organism number	MIC (%)		MBC (%)	
		*M. longifolia*	CHX	*M. longifolia*	CHX
*Streptococcus mutans*	PTCC1683	3.12	0.39	6.25	0.78
	Strain 1	3.12	0.39	6.25	0.78
	Strain 2	3.12	0.39	6.25	0.78
	Strain 3	6.25	0.39	12.5	0.78
	Strain 4	3.12	0.39	6.25	0.78
	Strain 5	3.12	0.39	6.25	0.78
*Streptococcus sobrinus*	PTCC27607	6.25	0.78	6.25	0.78
	Strain 1	6.25	0.78	12.5	0.78
	Strain 2	6.25	0.78	12.5	1.56
	Strain 3	3.12	0.78	6.25	0.78
	Strain 4	12.5	0.78	12.5	0.78
	Strain 5	6.25	0.78	12.5	1.56
*Lactobacillus*	PTCC1643	3.12	1.56	3.12	1.56
	Strain 1	3.12	1.56	6.25	1.56
	Strain 2	3.12	0.78	6.25	3.12
	Strain 3	3.12	0.78	6.25	3.12
	Strain 4	3.12	1.56	6.25	3.12
	Strain 5	3.12	1.56	3.12	1.56

The results showed that the antibacterial effect of *M. longifolia* essential oil had a similar effect on *S. mutans* and *Lactobacillus*, which was greater than the effect on *S. sobrinus*. *Lactobacillus strain* (*PTCC1643*) and *S. sobrinus strain* (*PTCC27607*) had the same MIC and MBC (3.12 and 6.25). In strains No. 3 of *S. mutans* and strains No. 3 and 4 of *S. sobrinus*, greater differences in MIC and MBC of *M. longifolia* essential oil and chlorhexidine were observed compared to other strains of *S. mutans* and *S. sobrinus*. In other samples, no significant difference was observed between standard strains and strains isolated from saliva. In fact, lactobacilli showed similar results in both strains.

### Statistical analysis

The results of a comparative study of the mean MIC and MBC in the group of *M. longifolia* and chlorhexidine essential oils are given in Table 2. Results of the t-test of the two dependent samples demonstrated significance level less than 0.05 in all groups. This is interpreted that a significant difference between *M. longifolia* and chlorhexidine exist in all groups.

**Table 2:** Comparison of mean differences of *M. longifolia* and chlorhexidine in minimum inhibitory concentration (MIC) and minimum bactericidal concentration (MBC) groups and standard strains microorganisms, results of paired t-test

	Pairs	Mean Difference	Std. Deviation	Paired Differences	95% Confidence Interval of the Difference		t-test	df	Sig. (2-tailed)
				Std. Error Mean	Lower	Upper			
Pair 1	MEO-MIC-SM-CHX-MIC-SM	3.251	1.277	.521	1.910	4.592	6.233	5	.002
Pair 2	MEO-MIC-SS-CHX-MIC-SS	5.990	3.073	1.254	2.764	9.215	4.774	5	.005
Pair 3	MEO-MIC-LB-CHX-MIC-LB	1.820	.402	.164	1.397	2.242	11.068	5	.000
Pair 4	MEO-MBC-SM-CHX-MBC-SM	6.511	2.551	1.041	3.833	9.189	6.251	5	.002
Pair 5	MEO-MBC-SS-CHX-MBC-SS	9.376	3.046	1.243	6.179	12.573	7.540	5	.001
Pair 6	MEO-MBC-LB-CHX-MBC-LB	2.866	1.178	.481	1.629	4.103	5.957	5	.002

MEO: Mentha Longifolia Essential Oil, SM: Streptococcus Mutans, SS: Streptococcus Sobrinus, LB: Lactobacillus

### Identification of components

**Figure 1:**
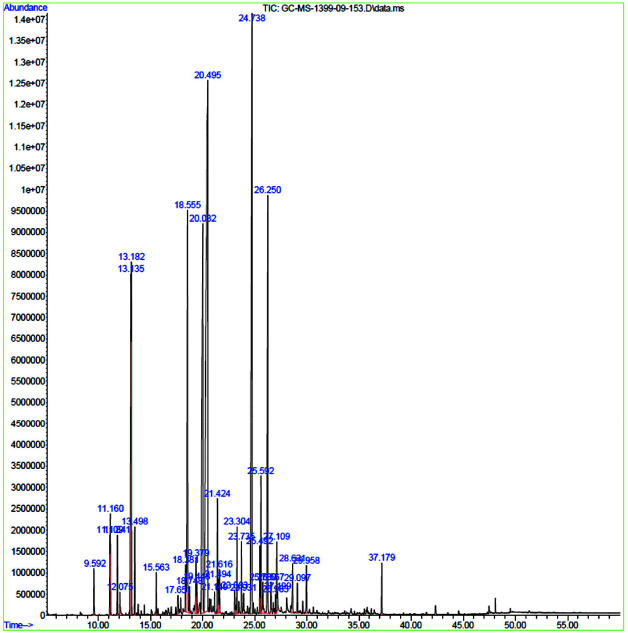
Gas chromatogram of the essential oil of *Mentha longifolia (L.)* Hudson. The peak numbers correspond to the numbers of the compounds listed in Table 3

The gas chromatogram obtained from this *M. longifolia* essential oil can be seen in Figure 1. The chemical components were detected by GC-MS technique. Identification of the constituents was based on the retention time (Rt) and computer matching against the spectra library Wiley 7 and NIST 08. A total of 38 compounds was identified, representing 99.99% of the extracted essential oil from the aerial parts of *M. longifolia* (Table 2). Their retention times and percentage compositions are given in Table 3. The major components were Piperitone Oxide (27.59%), trans-Caryophyllene (14.55%), and 2-Cyclohexene-1-one (12.24%).

**Table 3:** Chemical composition of the *M. longifolia* essential oil

Components	R_t_ (min)	% Rate
Alpha-pinene	9.952	0.56
Sabinene	11.108	0.90
2 Beta-pinene	11.160	1.18
beta.-Myrcene	11.842	0.89
dl-Limonene	13.136	6.31
1'8-Cineole	13.183	3.46
Linalool	15.561	0.53
Beta.-Myrcene	17.653	0.42
Alpha.Terpineo	l18.382	0.99
Neodihydrocarveo	l18.557	7.14
Trans-(+)-carveol	19.379	0.94
2-Cyclohexen-1-one	20.031	12.24
Piperitone Oxide	20.492	27.59
2-Cyclohexen-1-one, 2-hydroxy-3-	21.494	0.41
Piperitone oxide	23.301	1.09
Beta.bourbonene	23.733	0.89
trans-Caryophyllene	24.735	14.55
alpha.-Humulene	25.493	0.85
trans-.beta.-Farnesene	25.592	1.69
Linalool L	25.778	0.58
Germacrene-D	26.251	7.54
Naphthalene	26.985	0.21
Disugran	27.107	1.11
delta.-Cadinene	27.201	0.24
Other components		7.68
Total		99.99

Compounds listed in order of elution from a TRB Wax capillary column Retention time (as minutes)

## Discussion

Plant-derived antimicrobial compounds kill bacteria by mechanisms different from antibiotics, and this difference is clinically important in the treatment of infections caused by resistant microbial strains [ 23 ].

*M. longifolia* has a wide range of antimicrobial activities against various bacteria, yeasts, insects, and other organisms. Previous study has reported that *M. longifolia* essential oil has more antimicrobial activity than hydro alcoholic extract [ 9 ]. In 2016, Raeisi *et al*. [ 24 ], evaluated the effects of *Mentha Piperita* (*M. Piperita*) and NaCl on *S. mutans*, and found that *M. Piperita* hydro alcoholic extract had no antibacterial properties against *S. mutans* in agar and in disk diffusion methods, whereas the most effective concentration of NaCl on the bacterium was 5-5.5%. Limited studies have been performed to evaluate the effects of the essential oil of this plant on cariogenic microorganisms; therefore, this study aimed to investigate the antibacterial effect of *M. longifolia* essential oil on three cariogenic microorganisms; *S. mutans*, *S. sobrinus*, and *Lactobacillus* and to analyze the compounds this plant contains.

The results of the present study purport that the MIC rate for *S. mutans* was 3.12%, for *S. sobrinus* was 6.25% and for *Lactobacillus* was 3.12%.

Al-Bayati *et al*. [ 25 ] reported the MIC for *Staphylococcus aureus* and *S. mutans* was 6.15 and whilst that for *Enterococcus faecalis*, *Streptococcus pyogenes* and *Lactobacillus* acidophilus was 3.12µg/ml. The researchers found that the isolation and purification of antimicrobial agents from *M. longifolia* leaves could help treat sore throats and irritating mucosal lesions. Golestannejad *et al*. [ 26 ] in 2018 compared the antibacterial activity of Foeniculum vulgare Mill, Mentha arvensis, and *M. piperita* essential oils against *S. mutans* and discovered that Foeniculum vulgare essential oil has the lowest MIC and MBC on *S. mutans*, 8.4, and 14.9μg/ml respectively. They recorded MIC of 10.5 μg/ ml and MBC of 16.3μg/ml for *M. piperita* essential oil. The difference in values could be due to plant types and the composition of phytochemicals (active ingredients in medicinal plants). In a study by Kermanshah *et al*. [ 27 ], bactericidal and inhibitory effects of hydro alcoholic extracts of Salvia officinalis, *M. longifolia*, Achillea millefolium, and Pimpinella anistun on AT88 bacterium Lactobacillus rhamnosus (ATCC: 7469, PTCC: 1637) and *Actinomycosis viscose* (ATCC: 15987) were corroborated. The MIC of *M. longifolia* was 12.5µg/ml for *S. mutans*, 3.12µg/ml for *Lactobacillus* and 100µg/ml for Actinomycosis. The differences between this study and the present study could be due to the extract and the type of strains used. In addition, the concentration range of the extracts was 0.18-100µg/ml, while in the present study; the concentration range of the essential oil was 0.50µg/ml. In 2016, Ghasemi *et al*. [ 28 ] found that the MIC and MBC of *M. longifolia* extract for *S. mutans*, *Lactobacillus rhamnosus*, and *Actinomycosis viscous* were 110 and 165, 80 and 120, and 450 and 650μg/μl, respectively. The main constituents of *M. longifolia* in that study were pulegone extract (31.78%), 1,8-cineole (15.99%) and menthofuran (11.25%). They observed these compounds inhibit bacterial growth and could be used as a cost-effective medicinal alternative that is readily accessible. The difference in these results compared to the present study is the different types of compounds and active substances in both studies (extract compared to essential oil).

The essential oil in the present study is perceived to have a greater antibacterial effect against *S. mutans* and *Lactobacillus* microorganisms. The literature did not show any results for studies on the antibacterial effect of *M. longifolia* essential oil on *S. sobrinus*.

The results of GC/MS analysis of *M. longifolia* essential oil composition showed that the highest composition of *M. longifolia* essential oil was Piperitone oxide (27.59%), trans-Caryophyllene (14.55%), 2-cyclohexan-e-1-one (12.24%), and germacrene D (7.54%).

A 2002 study by Oumzil *et al*. [ 29 ] demonstrated antibacterial properties of Piperitone oxide against bacteria such as Staphylococcus aureus, Staphylococcus aureus, and Klebsiella pneumonia. In 2017, Azizan *et al*. [ 30 ] examined the composition and antibacterial activity of the essential oils of Orthosiphon stamineus Benth and Ficus deltoidea Jack against pathogenic bacteria including *Enterococcus faecalis*, *S. mutans*, and *Streptococcus Mitis*. Both essential oils had antibacterial activity against gram-positive and gram-negative oral bacteria and this could be attributed to the presence of active compounds such as trans-Caryophyllene, alpha-Humulene, eugenol and germacrene-D. The essential oil used in the present study has also contained trans-Caryophyllene (14.55%), alpha-Humulene (0.85%), and germacrene-D (7.54%).

 In a study of *M. longifolia* essential oil by Gulluce *et al*. [ 31 ], antibacterial activity against 16 species of bacteria, including Salmonella enteritidis, Staphylococcus epidermis, Bacillus megatherium, and 15 species of fungi, including Aspergillus flavus, Fusarium oxysporum, and Trichophyton were reported. The MIC range of susceptible bacteria was 15.62-125µg/ml. The most essential constituents of the oils were Cispiperitone epoxide (18.4%), Pulegone (15.5%), and Piperitone oxide (14.7%), and these accounted for the antibacterial function of the essential oil. In the study by Akhbar *et al*. [ 32 ], the main components of *M. longifolia* essential oil were decarvone and limonene. In another study, pulegone has been reported as the main constituent of the essential oil [ 33 ], but pulegone was not found in the essential oil of *M. longifolia* in the current study. The major polyphenol in methanolic leaf extracts of *M. longifolia* was rosmarinic acid, based on the study done by Elansary HO. *et al*. [ 34 ]. 

The composition of essential oil of one plant species may differ from that of the same species due to different regional conditions, which may be related to differences in the harvest season, times spent in essential oil extraction, differences in geographical areas, and even differences in the parts of the plant [ 16 , 35 ]. Bakkali *et al*. [ 36 ] found that compounds such as limonene, linalool, di-limonene, gamma-terpinene, p-cymene, alpha-pinene, and alpha-Terpineol have relatively strong antimicrobial activity. In the Pattnaik *et al*.’s [ 37 ] study, the antibacterial and antifungal activity of five aromatic compounds in plant essential oils were examined, of which linalool was identified as the most effective antibacterial compound with an ability to inhibit the growth of 17 bacterial strains (including gram-positive and gram-negative cocci and gram-negative radii). Naphthalene also has antibacterial and antifungal properties against various human pathogens [ 38 ]. The essential oil of the present study also contained alpha-Terpineol (0.99%), alpha-pinene (0.56%), linalool (0.53%) and naphthalene (0.21%). Although the main and predominant compounds of essential oils are considered as the main antibacterial agents, few studies have demonstrated the possibility that compounds with lower percentages could have a synergistic effect with other effective active compounds [ 39 ].

In 2007, Celiktas *et al*. [ 40 ] reported that essential oils exert their antibacterial effect by altering the structure and action of cell membranes. The altered cell membranes swell and have reduced activity which eventually leads to cell death. Thus, in the present study, the main reason for the antimicrobial effect of the essential oil *M. longifolia* on the tested bacteria can be attributed to this reason.

From the results of the present study, it can be said that *M. longifolia* has an antimicrobial effect on oral microbes. Since chlorhexidine mouthwash can cause discoloration and microbial imbalance [ 41 ], and amoxicillin, which is an antibiotic of choice in dentistry, can cause bacterial resistance [ 42 ], there is a need to introduce a substance that does not have these characteristics. The efficiency of *M. longifolia* essential oil against cariogenic microorganisms was confirmed in this study. However, a clinical study is recommended due to variable factors in the oral environment and the difference between the oral environment and the laboratory enviroment [ 43 ].

## Conclusion

The results of the present study showed that the MIC for *S. mutans* was 3.12%, *S. sobrinus* was 6.25%, and Lact-obacillus was 3.12%. The highest composition of *M. longifolia* essential oil is Piperitone oxide, Trans-Caryophyllene, 2-cyclohexane-1-one. Results from this study showed that *M. longifolia* essential oil had a growth inhibitory and lethal effect on all three cariogenic microorganisms, although this effect was less than chlorhexidine.

## Acknowledgement

This study was a part of a thesis and research project, which was supported and funded by Babol University of Medical Sciences.

## Conflict of Interest

The authors declare that they have no conflict of interest.
